# Friend or Foe: Factor XII Deficiency Discovered Incidentally during Management of NSTEMI

**DOI:** 10.1155/2023/5926340

**Published:** 2023-06-29

**Authors:** Patrick J. Beck, John Benfield, Joshua Morales

**Affiliations:** ^1^Virginia Tech Carilion School of Medicine, 2 Riverside Circle, Roanoke, VA 24016, USA; ^2^Department of Internal Medicine, Carilion Roanoke Memorial Hospital, 1906 Belleview Ave SE, Roanoke, VA 24014, USA; ^3^Blue Ridge Cancer Care, 2013 S Jefferson St, Roanoke, VA 24014, USA

## Abstract

Factor XII (FXII) deficiency is a rare coagulopathy that typically goes undiagnosed due to the lack of abnormal bleeding or thrombosis. However, the accompanying prolonged activated partial thromboplastin time (aPTT) can create difficulties with maintaining therapeutic anticoagulation in the setting of acute coronary syndrome (ACS). Here, we present the case of a 52-year-old man presenting with chest pain and diagnosed with an NSTEMI but also found with a prolonged baseline aPTT ultimately secondary to FXII deficiency. Here, we discuss the diagnostic work-up of an isolated prolonged aPTT to identify possible etiologies, such as FXII deficiency, and ultimately inform ACS management.

## 1. Introduction

Effective heparin anticoagulation is essential in the management of acute coronary syndrome (ACS); however, it can be difficult to achieve and maintain. Subtherapeutic heparin levels increase the risk for ACS sequelae including recurrent infarction, thromboembolic events during percutaneous coronary intervention (PCI), and death [1]. Conversely, life-threatening bleeding can occur if heparin is supratherapeutic, especially when combined with dual-antiplatelet therapy (DAPT) [2]. Therefore, heparin dosing is commonly guided by regular measurements of the activated partial thromboplastin time (aPTT) to ensure therapeutic levels [3]. Some patients with ACS may also demonstrate a prolonged aPTT at baseline, necessitating identification of the underlying etiology to quantify bleeding risk before proceeding with standard-of-care interventions. Thus, it is important to understand how to investigate a prolonged aPTT in the setting of ACS to inform potential limitations for anticoagulation, DAPT, and cardiac catheterization and properly manage these patients.

## 2. Case Presentation

A 52-year-old man with a past medical history of hypertension, hyperlipidemia, and type 2 diabetes mellitus presented to the emergency department for lower chest pain of six days duration radiating to his left arm with moderate shortness of breath on exertion. His admission EKG revealed *Q*-waves in the anterior and lateral leads without significant ST-segment changes, while serum troponins were elevated at 5.28 ng/mL. Due to the suspicion for non-ST-elevation myocardial infarction (NSTEMI), baseline labs were drawn (Table 1), medications, including a heparin drip and DAPT with aspirin and clopidogrel, were initiated, and he was admitted for further management.

Approximately one hour into the admission, his baseline aPTT was found to be >180 seconds, while INR was within normal limits. Further questioning revealed no significant personal or family bleeding history, and he denied taking anticoagulants prior to admission. Past surgical history was remarkable for a distant appendectomy and two toe amputations, for all of which he denied bleeding complications. Nonetheless, his heparin was stopped in anticipation for PCI, which revealed >50% stenosis of the middle left anterior descending artery and branches of both the left circumflex and right coronary arteries. He recovered well without abnormal intraoperative bleeding, but later in the day suffered a ventricular fibrillation cardiac arrest requiring three rounds of chest compressions before successful cardioversion. He proceeded to have two episodes of pulseless monomorphic ventricular tachycardia that evening again with successful cardioversion and was started on lidocaine and amiodarone drips. Over concern for ongoing ischemia, emergent PCI with aspiration thrombectomy was pursued the following morning. Two drug-eluting stents were placed in the left anterior descending artery, while one stent was placed in each obtuse marginal branch of the left circumflex artery. Following recovery, the decision was made to resume the heparin drip given his negative personal and family bleeding history, his lack of excessive or abnormal bleeding following both PCI procedures, and the need to prevent reinfarction and PCI-related thromboses in an already critically ill patient. Heparin drip rate was guided by antiactivated factor X (anti-Xa) levels, which helped maintain heparin in the therapeutic range (Table 1). DAPT with aspirin and clopidogrel was also continued shortly thereafter. Despite heparin and DAPT onboard, all arterial catheter sheaths were removed uneventfully, and he demonstrated no signs of abnormal postoperative bleeding.

Following the stabilization of his condition, the patient's isolated prolonged aPTT on admission was investigated (Table 2). The activated clotting time was elevated at 714 seconds, while an initial mixing study drawn while the heparin drip was active did not correct the aPTT. While it was suspected that heparin therapy was responsible for this result, lupus anticoagulant, cardiolipin antibody, and beta-2-glycoprotein I antibody titers were drawn and resulted negative. A repeat mixing study was drawn after brief heparin cessation, and the addition of normal plasma corrected the aPTT. Factor activity levels drawn simultaneously revealed undetectable factor XII (FXII) activity, and the patient was diagnosed with a severe FXII deficiency. Following medical optimization, he was discharged to a skilled nursing facility for rehabilitation. At subsequent follow-up visits, he denied bleeding events despite continuing DAPT for >1 year.

## 3. Discussion

In this case, our patient's isolated prolonged aPTT was ultimately secondary to a severe FXII deficiency, which did not contraindicate the use of heparin as FXII deficiency does not increase the risk for bleeding [4]. The prolonged aPTT did, however, make heparin maintenance more difficult, requiring the measurement of anti-Xa levels to inform heparin dosing [5]. Some studies have argued that FXII deficiency can paradoxically increase the risk for thrombosis, but many too readily cast aside the influence of established thrombotic risk factors such as fracture and prolonged immobilization [6], pregnancy [7], or surgical operations [8]. Moreover, no difference in thromboembolism rates was observed between patients with severe FXII deficiency and unaffected family members in a 20-year prospective cohort study, although the study sample size was low and there were few thromboembolic events overall [9]. We mention this to assert that despite some contradictory evidence in the literature, our patient's NSTEMI was most likely due to his cardiovascular disease risk factors, including sex, age, hypertension, hyperlipidemia, and type 2 diabetes mellitus, rather than his FXII deficiency.

While patients with FXII deficiency and ACS can be managed essentially the same as non-FXII-deficient patients, other causes of isolated prolonged aPTT do significantly impact ACS management, necessitating a proper diagnostic work-up to identify the underlying etiology. A thorough personal and family bleeding history should be collected to assess patient risk. Hemophilia A, B, and C are hereditary coagulopathies of the intrinsic clotting cascade characterized by the deficiencies of FVII, FIX, and FXI, respectively. Von Willebrand disease is another hereditary coagulopathy that can present with an isolated prolonged aPTT, as von Willebrand factor deficiency reduces the activity of FVIII in plasma [10]. In contrast with FXII deficiency, these coagulopathies typically present with recognizable patterns of easy and abnormal bleeding from a young age, which can make the management of ACS challenging. However, specific recommendations have been identified to maximize ACS treatment and minimize bleeding risk in these patients, including replacement therapy during and after PCI, specific stent choices, DAPT with clopidogrel and aspirin for as short duration as possible, and mandatory anticoagulation with unfractionated heparin given its short half-life and reversibility [11].

In a patient with a negative bleeding history, a thorough medication history should next be obtained. The use of oral anticoagulants, while typically causing both aPTT and PT/INR prolongation, should be assessed, including vitamin K antagonists (warfarin) and novel oral anticoagulants (NOAC) such as the direct factor Xa inhibitors (apixaban, rivaroxaban) and direct thrombin inhibitors (bivalirudin, argatroban, and dabigatran). Additionally, the use of herbal medicines containing coumarins, natural compounds similar to warfarin, such as chamomile, fenugreek, red clover, or ginseng should be assessed [12]. Patients taking warfarin with a therapeutic INR do not require additional peri-PCI anticoagulation, while patients on NOACs should be transitioned to heparin as their peri-PCI efficacy mixed [1]. Patients on chronic anticoagulation with warfarin or NOACs should complete single antiplatelet therapy with clopidogrel to reduce bleeding events without increasing the risk of thrombosis [13].

If the patient's isolated prolonged aPTT is not explained by a historical finding, and the activated clotting time (ACT) is also prolonged, then a mixing study should be ordered to assess for correction of the prolonged aPTT with the addition of normal plasma to the patient's blood. If the aPTT corrects, it suggests the deficiency or dysfunction of one or more intrinsic pathway clotting factor, which can be differentiated by measuring the individual factor activity levels. If the patient lacks significant bleeding history, but their aPTT corrects on the mixing study, FXII deficiency is highly likely. However, if the aPTT does not correct, it suggests etiologies including (1) therapy with or sample contamination from heparin; (2) antiphospholipid antibodies (APA) such as lupus anticoagulant, cardiolipin antibodies, or beta-2-glycoprotein I antibodies; or (3) an acquired factor inhibitor. Patients positive for APAs should be maintained on anticoagulation and DAPT, as these antibodies can prolong the aPTT and ACT, but produce a paradoxical hypercoagulable state *in vivo* [14]. Conversely, anticoagulation should be held in patients with an acquired inhibitor as this can result in significant bleeding mimicking hemophilia. Acquired inhibitors are autoantibodies against a specific clotting factor, most commonly FVIII, that can take weeks to resolve with immunosuppressive therapies such as glucocorticoids, rituximab, or intravenous immunoglobulins [15]; thus, patients can receive prophylaxis with a bypass agent or recombinant clotting factor to transiently improve clotting function for PCI if indicated [16].

In conclusion, this case illustrates the diagnostic and management complexity of a patient presenting simultaneously with ACS and an isolated prolonged aPTT, as anticoagulation, DAPT, and PCI are critical pieces of ACS management, but carry risks of bleeding that can become accentuated in coagulopathic patients.

## Figures and Tables

**Table 1:** Baseline coagulative laboratory values and heparin dosing.

Test	Patient's value	Reference range
aPTT (sec)	>180.0	24.0–37.0
INR	1.1	0.9–1.1
Heparin anti-Xa (IU/mL)	0.34	0.30–0.50

INR, international normalized ratio; aPTT, activated partial thromboplastin time.

**Table 2:** Workup of baseline isolated prolonged aPTT.

	Test	Patient's value	Reference range
Heparin onboard	Mixing study	aPTT does not correct with normal plasma	
	Lupus anticoagulant	Negative	Negative
	Cardiolipin antibodies	Negative	Negative
	Beta-2-glycoprotein I antibodies	Negative	Negative

Postheparin cessation	Activated clotting time (sec)	714	74–137
	Heparin anti-Xa (IU/mL)	<0.10	0.30–0.70
	Mixing study	aPTT corrects with normal plasma	
	Factor II activity (%)	92	70–120
	Factor VIII activity (%)	342	60–150
	Factor IX activity (%)	143	60–150
	Factor X activity (%)	63	70–120
	Factor XI activity (%)	84	60–150
	Factor XII activity (%)	<13	60–150

## Data Availability

All data supporting the conclusions of this case are available to the readers within the body of the publication.
